# Antibacterial, anti-inflammatory and antioxidant activities of Mahanintangtong and its constituent herbs, a formula used in Thai traditional medicine for treating pharyngitis

**DOI:** 10.1186/s12906-021-03274-6

**Published:** 2021-03-30

**Authors:** Bhanuz Dechayont, Pathompong Phuaklee, Jitpisute Chunthorng-Orn, Thana Juckmeta, Onmanee Prajuabjinda, Kitrawee Jiraratsatit

**Affiliations:** 1 Department of Applied Thai Traditional Medicine, Faculty of Medicine, Thammasat University, Pathum Thani, 12120 Thailand; 2 Faculty of Public Health, Thammasat University, Pathum Thani, 12120 Thailand

**Keywords:** *Streptococcus pyogenes*, Nitric oxide (NO), Tumor necrosis factor α (TNF-α), Interleukin 6 (IL-6), Thailand National List of essential medicines, Pharyngitis

## Abstract

**Background:**

Mahanintangtong is listed in the Thailand’s National List of Essential Medicines (NLEM). It is used to treat non-specific fevers and illnesses such as pharyngitis and chickenpox. In this study, we investigated the biological activities of the different medicinal plants used in the Mahanintangtong formula.

**Methods:**

The plant materials were extracted by maceration and decoction. Antimicrobial activity, assessed by disc diffusion method, the minimum inhibitory concentration (MIC), and minimum bactericidal concentration (MBC) were compared with commercially available standard antibiotics. To elucidate the anti-inflammatory mechanisms, inhibition of nitric oxide (NO), tumor necrosis factor α (TNF-α), and interleukin 6 (IL-6) production was tested by Griess and ELISA techniques. Antioxidant activity was measured by ABTS and DPPH scavenging assays.

**Results:**

The extracts with the best antimicrobial activities were carbonized *Tectona grandis* showing against *Streptococcus pyogenes*, *Staphylococcus aureus*, Methicillin-resistant *Staphylococcus aureus* (MRSA) and *Pseudomonas aeruginosa*. The ethanol extract of *Dracaena loureiroi* wood exhibited the highest NO and IL-6 inhibitory activity with IC_50_ values of 9.42 ± 1.81 and 12.02 ± 0.30 μg/mL, respectively. The ethanol extract of *Pogostemon cablin* had the highest TNF-α inhibitory with IC_50_ values of 10.68 ± 0.02 μg/mL. In anti-free radical testing, the ethanol extract of *D. loureiroi* displayed high antioxidant activity by both ABTS and DPPH assays.

**Conclusion:**

The ethanol extracts from carbonized *T. grandis* and Mahanintangtong showed good antimicrobial activity, especially against *S. pyogenes*, and good anti-inflammatory activity. These findings are relevant to the pathogenesis of pharyngitis and justify additional studies to see if Mahanintangtong could have clinical utility.

## Background

Pharyngitis is inflammation of the pharynx that is most often caused by viruses but may also be caused by bacteria, notably, *Streptococcus pyogenes* (group A Streptococcus [GAS]) [1]. One of the first defenses against infection is a host-mounted inflammatory response involving a variety of cytokines and chemokines which both trigger and/or enhance the specific inflammatory response [2].

Inflammation is a complex process that is coordinated by cytokines and immune signalling molecules [3]. The concept of the immune signalling which induces fever during infection and inflammation has been widely accepted. The well-identified pathways of inflammation are either activated by lipopolysaccharide (LPS) or by key cytokines such as IL-6, IL-1β, TNF-α and others. Other putative endogenous mediators like lipocortin-1 are also involved in fever [4, 5].

LPS and proinflammatory cytokines can catalyse the formation of small signalling molecules such as nitric oxide (NO), which plays a key role in the pathogenesis of inflammation. NO has an anti-inflammatory effect under normal physiological conditions but it is a pro-inflammatory mediator that induces inflammation due to its over production in acute inflammatory states and physiological stress [6]. Therefore, finding the inhibitors which are able to block inflammatory cytokines are important for reducing the inflammatory response.

For treating common bacterial infections like a streptococcal sore throat, penicillin is commonly recommended to decrease symptoms and to prevent subsequent complications and the spread of infection [7, 8]. However, drug side effects and microbial resistance are two reasons for the renewed interest in traditional herbal medicines. The use of plant-based formulations is potentially a viable option for reducing the side effects associated with conventional antibiotic treatment [9, 10] as well as increase the number of compounds that could be used for treating infections or acting synergistically with current therapies [11].

Thailand’s National List of Essential Medicines (NLEM), a knowledge repository of Thai traditional herbal medicine, includes information on single herbs and herbal combinations used for treating pharyngitis. The herbal combinations are typically composed of herbs with complimentary properties. Mahanintangtong is a herbal combination described in NLEM for treating non-specific fevers as well as pharyngitis and chickenpox. The formula consists of several herbal plants: carbonized seeds of *Entada rheedii* Spreng, carbonized seeds of *Spondias pinnata* (L.f.) Kurz, carbonized pulp of *Sapindus rarak* DC, carbonized wood of *Calamus caesius* Blume, carbonized wood of *Tectona grandis* L.f., *Dracaena loureiroi* Gagnep, *Myristica fragrans* Houtt, and the leaves of *Pogostemon cablin* (Blanco) Benth, *Tiliacora triandra* (Colebr.), Diels, Chinese ink (Inkstick), and Shell of *Monetaria moneta* (Money cowry) [12].

Previous phytochemical and pharmacological studies have shown that plant extracts have anti-inflammatory properties but no specific work has been done on the Mahanintangtong formula. Therefore, we tested extracts from Mahanintangtong for antibacterial, anti-inflammatory and antioxidant properties.

## Methods

### Chemicals and reagents

All the chemicals and reagents used were of analytical grade. 2, 2–Diphenyl-1-picrylhydrazyl (DPPH), and butylated hydroxytoluene (BHT) were purchased from Fluka, Germany. Acetic acid was purchased from Merck, Germany. 2,2′-Azino-bis (3-ethylbenzo-thiazoline-6-sulfonic acid) diammonium salt, 6-hydroxy-2,5,7,8-tetramethylchroman-2-carboxylic acid, potassium persulfate, dimethyl sulfoxide, lipopolysaccharide from *E.coli* O55:B5 (LPS), phosphoric acid, N-(1-Naphthyl) ethylenediamine dihydrochloride, sulfanilamide,3-(4,5-Dimethyl-2-thiazolyl)-2,5-dipheyl-2H-tetrazolium bromide or thiazolyl blue tetrazolium bromide (MTT) were purchased from Sigma-Aldrich, USA. Dulbecco’s modified eagle medium (DMEM), Foetal bovine serum (FBS), Trypan blue stain 0.4% and Trypsin-EDTA were obtained from Gibco, USA. Hydrochloric acid and isopropanol were obtained from RCI Labscan, Thailand; Phosphate-buffered saline (PBS) was provided by Biochrom, Germany and mouse TNF-α and IL-6 ELISA kits were purchased from ImmunoTools, Germany.

### Plant material and preparation of extracts

The plant materials were purchased from a licensed traditional medicine drug store, Charoensuk Osod in Nakorn Pathom, Thailand. The herbal materials were identified and authenticated by Department of Applied Thai Traditional Medicine, Thammasat University. A voucher specimen is kept at the Herbarium of the Faculty of Pharmaceutical Sciences, Prince of Songkla University, Thailand. A voucher specimen is shown in Table 1. Then the plant materials were crushed in to small pieces for the extraction process. The formula was prepared from 11 ingredients; its constituents are shown in Table 1.

**Table 1:** Yields (%w/w), plant name, part of plant used, voucher specimen and % in formulation of different components of the Mahanintangtong formula and its constituent herbs

Plant name	Family	Part used	Voucher specimen	Formulation (%wt)	Extracts type	% Yield
*Entada rheedii* Spreng. (carbonized)	Fabaceae	Seed	SKP 098/115 05 18 01	9.71	Ethanol	3.96
					Water	9.46
*Spondias pinnata* (L.f.) Kurz. (carbonized)	Anacardiaceae	Seed	SKP 009 19 16 01	9.71	Ethanol	6.20
					Water	1.95
*Sapindus rarak* DC. (carbonized)	Sapindaceae	Pulp	SKP 170 19 05 01	9.71	Ethanol	4.64
					Water	2.98
*Calamus caesius* Blume. (carbonized)	Arecaceae	Wood	SKP 137 03 03 01	9.71	Ethanol	0.58
					Water	4.20
*Tectona grandis* L.f. (carbonized)	Lamiaceae	Wood	SKP 095 20 07 01	9.71	Ethanol	0.84
					Water	0.32
*Dracaena loureiroi* Gagnep.	Asparagaceae	Wood	SKP 065 04 12 01	9.71	Ethanol	17.79
					Water	1.48
*Myristica fragrans* Houtt.	Myristicaceae	Wood	SKP 121 13 06 01	9.71	Ethanol	3.94
					Water	2.00
*Pogostemon cablin* (Blanco) Benth.	Lamiaceae	Leaves	SKP 095 16 03 01	9.71	Ethanol	7.47
					Water	12.70
*Tiliacora triandra* Diels	Menispermaceae	Leaves	SKP 114 20 20 01	9.71	Ethanol	3.94
					Water	6.54
Chinese ink	–	–	–	9.71	Ethanol	N/A
					Water	2.92
*Monetaria moneta* (Money cowry)	Cypraeidae	Shell	–	2.90	Ethanol	N/A
					Water	0.40
Mahanintangtong formula	–	–	–	–	Ethanol	4.98
					Water	4.12

*N/A* Not Applicable

Seeds of *Entada rheedii* and *Spondias pinnata*, pulp of *Sapindus rarak,* wood of *Calamus caesius* and *Tectona grandis* were carbonized following the Thai Traditional method: heated at 300 °C in clay pot for 24 h., and, then, as charcoal, for a further 6 h, before cooling at room temperature.

### Extraction method

#### Maceration method

This followed the method of Dechayont et al. [13]. The Mahanintangtong formula and its individual herb components were dried in an oven at 50 °C. The dried materials (50 g) were then extracted three times with 95% ethanol (EtOH) at room temperature (72 h) and filtered through Whatman No. 1 filter paper. The extracts were evaporated in a rotary evaporator and vacuum to dryness to give the crude ethanol extract.

#### Decoction method

Decoction was performed according to the method of Thai traditional medicine. The Mahanintangtong formula and the individual herbs (30 g) were boiled in distilled water for 15 min, cooled and then reboiled twice more. The aqueous mix was filtered through Whatman No. 1 filter paper and dried using a lyophilizer to give the crude water extract.

The ethanol and water extract yields were calculated as w/w and expressed as percentages (Table 1) and were stored at − 20 °C.

### Antimicrobial assay

#### Microbial strains

Cultures of the following microorganisms were used in the study: *Staphylococcus aureus* ATCC 25923; methicillin resistant *Staphylococcus aureus* (MRSA) ATCC 20651, *Streptococcus pyogenes* ATCC 19615, *Pseudomonas aeruginosa* ATCC 9097 and *Candida albicans* ATCC 90028.

### Disc diffusion method

The agar disc diffusion method was used to determine the antimicrobial activities of the extract, as described previously [14, 15]. Paper discs (6 mm diameter) were added to the extracts (conc. 5 mg/disc for ethanol extract, and conc. 1 mg/disc for water extract). The inoculum density was adjusted to 0.5 of the McFarland standard. Air-dried discs were placed on inoculated Mueller-Hinton agar (MHA) for *S. aureus*, MRSA and *P. aeruginosa*, MHA with 5% sheep blood for *S. pyogenes* and Sabouraud Dextrose agar (SDA) for *C. albicans*. Positive controls were amoxicillin, ampicillin, gentamicin and vancomycin, each at a concentration of 10 μg/disc. These plates were incubated at 37 °C for 24 h for bacteria and at 37 °C for 48 h for *C. albicans*. The zone of inhibition was calculated by measuring the diameter of the inhibition zone. The readings were taken in three different fixed directions in all 3 replicates and the mean value was tabulated.

### Determination of the minimal inhibitory concentration *(*MIC*)*

The minimal inhibitory concentration (MIC) of an antimicrobial agent is the lowest concentration of the antimicrobial agent that inhibits a given bacterial isolate from multiplying and producing visible growth in the test system [16]. Isolated colonies were prepared from 18 to 24 h cultures of *S. aureus*, MRSA, *S. pyogenes* and from 36 to 48 h cultures of *C. albicans* on an agar plate. Then the inoculum was adjusted with Mueller-Hinton broth (MHB) medium to 0.5 McFarland standard using a densitometer and the cultures were diluted 200 - fold. In sterile 96 - well microtiter plates, 50 μL of a plant extract was diluted 2-fold with broth (10 concentrations). Triplicate samples were used for each test concentration. After incubation for 16–18 h of *S. aureus*, MRSA, *S. pyogenes* and for 36–48 h cultures of *C. albicans* at 37 °C, 10 μL of resazurin solution was added in each well and incubated for 2 h. The lowest concentration value was determined in triplicate.

### Determination of the minimum bactericidal concentration (MBC)

Innocula from the wells that had no visible growth were transferred to the agar plates in quadrants and were incubated at 37 °C for 24 h and then examined for bacterial growth. The last quadrant (i.e. with the lowest concentration of plant extract) that showed no growth was taken as the MBC. Values were recorded as mg/ml and all experiments were performed in triplicate [17].

#### In vitro assay for anti-inflammatory assay

### Cell line

The inhibitory effects of nitric oxide (NO), TNF-α and IL-6 production were evaluated using murine macrophage like RAW 264.7 cells. The RAW 264.7 cell line was cultured in DMEM medium supplemented with 10% heated fetal bovine serum and 50 IU/mL penicillin and 50 μg/mL streptomycin. The cells were detached with trypsin-EDTA and diluted to a suspension in fresh medium.

### Assay for NO inhibitory effect

The evaluation of the NO inhibitory activity was performed in a manner similar to that described for cell viability [18]. The cells were seeded at a density of 1 × 10^5^ cells/well in 96-well microplates and incubated to adhere for 24 h at 37 °C in a humidified atmosphere containing 5% CO_2_. After that the medium was replaced with fresh medium containing a final concentration at 5 ng/mL of LPS. The adherent cells were treated with test samples at six concentrations to give a total volume of 200 μL/well and incubated for 24 h. Twenty-four hours later, the presence of nitrite was determined in cell culture media using Griess reagent at 570 nm. Inhibition (%) was calculated using the following equation and the IC_50_ value was calculated graphically using GraphPad 4.0 software in triplicate (Prism, USA).

1 $$ \mathrm{The}\%\mathrm{of}\ \mathrm{inhibition}=\left(\mathrm{Abs}.\mathrm{Control}-\mathrm{Abs}.\mathrm{Sample}\right)/\mathrm{Abs}.\mathrm{Control}\times 100 $$

### TNF-α and IL-6 inhibitory effect in RAW264.7 cells by ELISA

RAW 264.7 cells were incubated for 24 h with 5 ng/mL of LPS and various concentrations of extracts. After 24 h, supernatants were tested for the production of TNF-α and IL-6 quantified using an ELISA kit (ImmunoTools), following the manufacturer’s instructions; the optical density (OD) at 450 nm was determined on a plate reader [19]. The experiment compared potential with andrographolide as positive control which is a major active compound from the *Andrographis paniculata* plant.

### Assay for cytotoxicity – MTT assay

The cell viability of test sample was performed using the 3-(4,5-dimethyl-2-thiazolyl)-2,5-diphenyl-2H-tetrazolium bromide (MTT) colorimetric method. The RAW 264.7 macrophages were cultured in 96-well plates and allowed to grow at 37 °C in 5% CO_2_ for 24 h. Subsequently, the cells were tested with samples at six concentrations and, after 48 h, MTT solution (5 mg/mL of MTT in PBS) was added to the wells. Following 4 h of incubation, the medium was removed and 100 μL of isopropanol containing 0.04 M HCl was added to dissolve the formazan production in the cells. The OD of formazan solution was measured with a microplate reader at 570 nm [20]. The test simples were considered to be cytotoxic when the OD of the sample-treated group was less than 70% of that in the control (vehicle-treated) group. All determinations were carried out in triplicate. The percentage survival of the cell growth at each extract is calculated as:

2 $$ \mathrm{The}\%\mathrm{of}\ \mathrm{Survival}=\left(\mathrm{Abs}.\mathrm{Sample}/\mathrm{Abs}.\mathrm{Control}\right)\times 100 $$

### Antioxidant activity

#### DPPH radical scavenging assay

A stock solution of DPPH solution in absolute EtOH (6 × 10^− 5^ M) was freshly prepared and protected from light [17]. The herb extracts were prepared in a serial dilution (5 concentrations). The extracts 100 μL were individually added and mixed with an equal volume of DPPH solution in 96-well microplates and maintained in the dark at room temperature for 30 min. The absorbance was measured at 520 nm. The scavenging activity was calculated as a percentage inhibition in the formula (3) below:

3 $$ \mathrm{The}\%\mathrm{of}\ \mathrm{inhibition}=\left(\mathrm{Abs}.\mathrm{Control}\hbox{-} \mathrm{Abs}.\mathrm{Sample}\right)/\mathrm{Abs}.\mathrm{Control}\times 100 $$

The IC_50_ was calculated from a dose-response curve using GraphPad 4.03 software.

#### ABTS radical cation decolorization assay

The ABTS reagent was prepared by mixing ABTS 7 mM concentration with 2.45 mM potassium persulfate in deionized water and left in the dark at room temperature for 12–16 h [21]. The mixture reagent was diluted with deionized water to give an absorbance of 0.700 ± 0.020 at 734 nm. Serial dilutions of the herbal extracts were made in absolute EtOH for the ethanol extracts and distilled water for the aqueous extracts. ABTS reagent (180 μL) was mixed with 20 μL of sample solution at five concentrations and the absorbance was measured after exactly 6 min at 734 nm in 96-well microplates to determine the scavenging activity. All determinations were carried out in triplicate. The scavenging activity was calculated as a percentage inhibition in the formula (4) below.

4 $$ \mathrm{The}\ \mathrm{Percentage}\ \mathrm{of}\ \mathrm{inhibition}=\left(\mathrm{Abs}.\mathrm{Control}-\mathrm{Abs}.\mathrm{Sample}\right)/\mathrm{Abs}.\mathrm{Control}\times 100 $$

The IC_50_ was calculated from a dose-response curve using GraphPad 4.03 software.

### Statistical analysis

All the experiments were conducted in triplicate. Results are expressed as mean ± standard error of the mean.

## Results

### Preparation of extracts

The yield percentages of Mahanintangtong formula and its 11 components are shown in Table 1. The maximal extract yield by maceration was the wood of *D. loureiroi* (17.79%) whilst the highest yield by decoction was the leaves of *P. cablin* (12.70%).

#### Antimicrobial assay

The antimicrobial activities of ethanol and water extracts were tested on all the ATCC bacteria and *C. albicans* using the disc diffusion assay, which was assessed by the presence and absence of inhibition zones. The ethanol and water extracts were tested at 5 and 1 mg/disc, respectively. In the control tests, DMSO was shown to have no inhibitory effect on any of the bacteria tested. The positive controls were amoxicillin, vancomycin, ampicillin and gentamicin. Based on inhibition zone test results, the ethanol extract of *T. grandis* (carbonized), *S. pinnata* (carbonized), and Mahanintangtong formula produced moderate activity against *S. pyogenes*, *S. aureus* and MRSA (inhibition zone > 10 mm, Table 2) while all the extracts showed no significant inhibition zones against *C. albicans*.

**Table 2:** Antimicrobial activity against five microorganisms of Mahanintangtong formula and its constituent herbs (*n* = 3)

Plant name	Extracts type	Diameter of inhibition zone (mm.)^*^, MIC (μg/mL)^**^, MBC (μg/mL)^***^				
		*S. pyogenes*	*S. aureus*	MRSA	*P. aeruginosa*	*C. albicans*
*Entada rheedii* (carbonized)	Ethanol	11.00±0.00^*^, 0.039^**^, 0.039^***^	11.33±0.58^*^, 0.625^**^, 0.625^***^	11.21±1.73^*^, 5^**^, 5^***^	0^*^, 0.625^**^, 0.625^***^	0^*^, >5^**^, >5^***^
	Water	0^*^, >5^**^, >5^***^	0^*^, 0.625^**^, 0.625^***^	0^*^, 1.25^**^, 1.25^***^	0^*^, 0.625^**^, 2.5^***^	0^*^, >5^**^, >5^***^
*Spondias pinnata* (carbonized)	Ethanol	12.67±2.52, 0.039, 0.039^***^	11.33±0.58, 0.625, 0.625^***^	12.00±0.00, 0.625, 0.625^***^	8.33±0.58, >5, >5^***^	0, >5, >5^***^
	Water	0^*^, >5^**^, >5^***^	0^*^, 0.625^**^, 0.625^***^	0^*^, 0.625^**^, 0.625^***^	0^*^, 5^**^, >5^***^	0^*^, >5^**^, >5^***^
*Sapindus rarak* (carbonized)	Ethanol	10.33±1.52^*^, 0.039^**^, 0.039^***^	0^*^, 0.625^**^, 0.625^***^	0^*^, 0.625^**^, 0.625^***^	0^*^, 0.625^**^, 0.625^***^	0^*^, >5^**^, >5^***^
	Water	0^*^, >5^**^, >5^***^	0^*^, 1.25^**^, 1.25^***^	0^*^, 1.25^**^, 2.5^***^	0^*^, >5^**^, >5^***^	0^*^, >5^**^, >5^***^
*Calamus caesius* (carbonized)	Ethanol	0^*^, 0.3125^**^, 0.625^***^	0^*^, 2.5^**^, 2.5^***^	0^*^, >5^**^, >5^***^	0^*^, 0.625^**^, 2.5^***^	0^*^, >5^**^, >5^***^
	Water	0^*^, >5^**^, >5^***^	0^*^, 2.5^**^, 2.5^***^	0^*^, 5^**^, >5^***^	0^*^, 5^**^, >5^***^	0^*^, >5^***^, >5^***^
*Tectona grandis* (carbonized)	Ethanol	13.33±0.58^*^, 0.078^**^, 0.078^***^	13.00+0.00^*^, 0.625^**^, 1.25^***^	14.33±0.58^*^, 0.625^**^, 0.625^***^	0^*^, 0.625^**^, 0.625^***^	0^*^, >5^**^, 5^***^
	Water	0^*^, >5^**^, >5^***^	8.00±0.00^*^, 0.625^**^, 0.625^***^	10.33±1.53^*^, 2.5^**^, 2.5^***^	0^*^, 2.5^**^, 2.5^***^	0^*^, >5^**^, >5^***^
*Dracaena loureiroi*	Ethanol	12.33±0.58^*^, 0.156^**^, 0.156^***^	11.00±0.00^*^, 0.625^**^, 0.625^***^	10.33±0.58^*^, 0.3125^**^, 0.625^***^	0^*^, 1.25^**^, >5^***^	0^*^, >5^**^, >5^***^
	Water	0^*^, >5^**^, >5^***^	0^*^, 0.625^**^, 0.625^***^	0^*^, 0.625^**^, 0.625^***^	0^*^, 5^**^, >5^***^	0^*^, >5^**^, >5^***^
*Myristica fragrans*	Ethanol	10.33±1.15^*^, 0.625^**^, 0.156^***^	0^*^, 0.625^**^, 0.625^***^	0^*^, 0.625^**^, 0.625^***^	0^*^, 0.625^**^, >5^***^	0^*^, >5^**^, >5^***^
	Water	0^*^, >5^**^, >5^***^	0^*^, 0.625^**^, 0.625^***^	0^*^, 0.625^**^, 0.625^***^	0^*^, 1.25^**^, >5^***^	0^*^, >5^**^, >5^***^
*Pogostemon cablin*	Ethanol	10.33±1.15^*^, 0.039^**^, 0.039^***^	10.33±2.52^*^, 0.625^**^, 0.625^***^	11.67±1.53^*^, 5^**^, 5^***^	0^*^, 0.625^**^, 0.625^***^	0^*^, >5^**^, >5^***^
	Water	0^*^, >5^**^, >5^***^	0^*^, 0.625^**^, 0.625^***^	8.33±1.53^*^, 2.5^**^, 2.5^***^	0^*^, 1.25^**^, 1.25^***^	0^*^, >5^**^, >5^***^
*Tiliacora triandra*	Ethanol	8.33±0.58^*^, 0.078^**^, 0.078^***^	0^*^, 0.625^**^, 0.625^***^	8±0.00^*^, 5^**^, 5^***^	7±0.00^*^, 5^**^, >5^***^	0^*^, >5^**^, >5^***^
	Water	0^*^, >5^**^, >5^***^	0^*^, 0.625^**^, 0.625^***^	0^*^, 1.25^**^, 2.5^***^	0^*^, >5^**^, >5^***^	0^*^, >5^**^, >5^***^
Chinese ink (Inkstick)	Ethanol	N/A	N/A	N/A	N/A	N/A
	Water	0^*^, >5^**^, >5^***^	0^*^, 5^**^, >5^***^	0^*^, >5^**^, >5^***^	0^*^, >5^**^, >5^***^	0^*^, >5^**^, >5^***^
*Monetaria moneta* (Money cowry)	Ethanol	N/A	N/A	N/A	N/A	N/A
	Water	0^*^, >5^**^, >5^***^	0^*^, >5^**^, >5^***^	0^*^, 2.5^**^, >5^***^	0^*^, >5^**^, >5^***^	0^*^, >5^**^, >5^***^
Mahanintangtong formula	Ethanol	10±0.00^*^, 0.078^**^, >5^***^	10±0.00^*^, 0.625^**^, 0.625^***^	10±0.00^*^, 0.625^**^, 0.625^***^	0^*^, 0.625^**^, 0.625^***^	0^*^, >5^**^, >5^***^
	Water	0^*^, >5^**^, >5^***^	0^*^, 0.625^**^, 0.625^***^	0^*^, 0.625^**^, 2.5^***^	0^*^, >5^**^, >5^***^	0^*^, >5^**^, >5^***^
Amoxicillin	NT	35.00±0.00^*^, 0.016^**^, 0.0256^***^	NT	NT	NT	NT
Ampicillin	NT	NT	43.00±0.00^*^, 0.25^**^, 1.56^***^	NT	NT	NT
Gentamicin	NT	NT	15.00±0.00^*^, 0.195^**^, 0.195^***^	10.00±0.00^*^, >200^**^, >200^***^	12.00±0.00^*^, 0.195^**^, 0.39^***^	NT
Vancomycin	NT	15.00±0.00^*^, 0.39^**^, 0.39^***^	45.00±0.00^*^, 0.003^**^, 1.92^***^	16.00±0.00^*^, 0.78^**^, 1.56^***^	NT	NT

Means of three measurements + SEM (*n* = 3). Amoxicillin, Ampicillin, Gentamicin and Vancomycin were tested at 10 μg/disc. *NT* Not Test, *N/A* Not Applicab

The MIC and MBC were measured to assess the potency of Mahanintangtong formula and its component extracts using the microdilution assay. These methods confirmed the results obtained by the disc diffusion method. The ethanol extracts were the most active: *E. rheedii* (carbonized), *S. pinnata* (carbonized), *S. rarak* (carbonized) and *P. cablin* extracts inhibited *S. pyogenes* with a MIC and MBC of 0.039 mg/mL. All the ethanol and water extracts except Chinese ink and money cowry showed activity against *S. aureus* with MICs and MBCs of 0.625–2.5 mg/mL, respectively. Although the ethanol extract of *C. caesius* was potent against *S. aureus*, it did not inhibit MRSA. The highest MIC against MRSA was 0.3125 mg/mL from ethanol extract of *D. loureiri*. The ethanol extract of *E. rheedii* (carbonized), *T. grandis* (carbonized), *P. cablin* and Mahanintangtong formula (herbal mix) showed the highest MIC and MBC values of 0.625 mg/mL against *P. aeruginosa*. None of the ethanol extracts had inhibitory activity against *C. albicans*.

### Anti-inflammatory assay

The ethanol extract of *D. loureiroi* exhibited highest NO inhibitory activity with IC_50_ values of 9.42 ± 1.81 μg/mL. The IC_50_ for Mahanintangtong formula was higher at 28.03 ± 0.64 μg/mL.

The ethanol extract of *P. cablin* had the highest inhibitory activity against TNF-α production with an IC_50_ = 10.61 ± 0.04 μg/mL; values for the wood of *M. fragrans* and carbonized wood of *T. grandis* were 14.03 ± 2.12 μg/mL and 21.26 ± 0.40 μg/mL, respectively. IL-6 inhibition was dose-dependent in the range of 1–100 μg/mL of ethanol extract. *D. loureiroi* had the greatest inhibitory effect with an IC_50_ 12.02 ± 0.30 μg/mL (Table 3). Inhibition was less for the aqueous extracts.

**Table 3:** Anti-inflammatory activity by inhibitory effects on NO, TNF-α and IL-6 production in RAW264.7 cells of the Mahanintangtong formula and its constituent herbs (*n* = 3)

Plant name	Extracts type	IC_**50**_ ± SEM (μg/ml)			Cytotoxic activity
		NO	TNF-α	IL-6	% Survival (conc. 100 μg/mL)
*Entada rheedii* (carbonized)	Ethanol	> 100	> 100	> 100	79.74 ± 3.45
	Water	> 100	> 100	> 100	73.06 ± 2.83
*Spondias pinnata* (carbonized)	Ethanol	> 100	> 100	> 100	102.65 ± 4.72
	Water	> 100	> 100	> 100	88.23 ± 0.78
*Sapindus rarak* (carbonized)	Ethanol	66.78 ± 1.38	> 100	> 100	71.86 ± 3.09 (conc. 70 μg/mL)
	Water	> 100	> 100	> 100	95.58 ± 6.81
*Calamus caesius* (carbonized)	Ethanol	> 100	> 100	> 100	93.97 ± 3.99
	Water	> 100	> 100	> 100	107.91 ± 6.05
*Tectona grandis* (carbonized)	Ethanol	52.47 ± 3.58	21.26 ± 0.40	49.38 ± 1.44	97.57 ± 5.36
	Water	> 100	> 100	> 100	97.64 ± 1.88
*Dracaena loureiroi*	Ethanol	9.42 ± 1.81	55.27 ± 5.47	12.02 ± 0.30	92.49 ± 1.39
	Water	> 100	> 100	> 100	98.54 ± 0.92
*Myristica fragrans*	Ethanol	35.78 ± 2.22	14.03 ± 2.12	55.26 ± 4.50	96.14 ± 1.68 (conc. 50 μg/mL)
	Water	> 100	> 100	> 100	97.97 ± 0.20
*Pogostemon cablin*	Ethanol	> 100	10.68 ± 0.02	17.37 ± 0.78	95.45 ± 2.80
	Water	> 100	> 100	> 100	94.86 ± 3.43
*Tiliacora triandra*	Ethanol	> 100	82.22 ± 3.89	> 100	96.06 ± 23.11
	Water	> 100	> 100	> 100	92.05 ± 5.54
Chinese ink (Inkstick)	Ethanol	> 100	N/A	N/A	N/A
	Water	> 100	> 100	> 100	90.15 ± 6.62
*Monetaria moneta* (Money cowry)	Ethanol	> 100	N/A	N/A	N/A
	Water	> 100	> 100	> 100	93.98 ± 2.09
Mahanintangtong formula	Ethanol	28.03 ± 0.64	33.09 ± 0.22	17.20 ± 0.65	88.85 ± 0.56
	Water	> 100	> 100	> 100	98.56 ± 0.74
Andrographolide		1.797 ± 0.41	5.08 ± 3.48	9.10 ± 0.61	91.58 ± 2.87

Means of three measurements ± SEM (*n* = 3). % Survival was tested at 100, 70 or 50 μg/mL after 24 h incubation in RAW 264.7 cells

### Cytotoxicity

At concentrations of 50 and 100 μg/mL of ethanol and water extracts, there was no effect on cell viability. At all concentrations tested, there was more than 70% of cell viability.

### Antioxidant assay

Several assays have been frequently used to estimate antioxidant activity of crude plant extracts. The most used assays are ABTS and DPPH because they both involve H- or electron-donation to the antioxidant [22]. Moreover, both have excellent stability under certain conditions. High ABTS scavenging activity was seen with the ethanol and water extracts of *D. loureiroi* and the ethanol extract of *T. grandis* as well as the water extract of *S. pinnata* (carbonized) and *E. rheedii* (carbonized). IC_50s_ ranged from 8 to 10 μg/mL compared to the positive controls: Trolox = 4.71 ± 0.04 μg/mL and BHT = 5.66 ± 0.26 μg/mL (Table 4.). In the DPPH assay, the ethanol extract of *S. pinnata* (carbonized) showed the highest activity with IC_50_ of 5.72 ± 0.90 μg/mL compared to 14.87 ± 0.94 (BHT) and 5.02 ± 0.20 (Trolox, Table 4).

**Table 4:** Antioxidant activity of the Mahanintangtong formula and its constituent herbs (*n* = 3)

Plant name	Extracts type	IC_**50**_ ± SEM (μg/ml)	
		ABTS radical-scavenging assay	DPPH radical-scavenging assay
*Entada rheedii* (carbonized)	Ethanol	22.60 ± 0.86	21.15 ± 3.72
	Water	10.29 ± 1.19	37.34 ± 0.54
*Spondias pinnata* (carbonized)	Ethanol	14.08 ± 2.49	5.72 ± 0.90
	Water	10.18 ± 1.05	28.64 ± 1.25
*Sapindus rarak* (carbonized)	Ethanol	> 100	> 100
	Water	94.14 ± 6.71	> 100
*Calamus caesius* (carbonized)	Ethanol	54.05 ± 0.75	58.93 ± 2.85
	Water	26.16 ± 1.16	> 100
*Tectona grandis* (carbonized)	Ethanol	10.13 ± 0.77	18.59 ± 2.65
	Water	24.81 ± 2.20	30.12 ± 1.79
*Dracaena loureiroi*	Ethanol	8.38 ± 0.95	16.95 ± 1.43
	Water	10.92 ± 1.23	19.65 ± 0.30
*Myristica fragrans*	Ethanol	49.69 ± 2.02	19.76 ± 0.98
	Water	24.21 ± 1.13	30.99 ± 1.33
*Pogostemon cablin*	Ethanol	63.50 ± 3.88	17.81 ± 1.15
	Water	35.09 ± 0.96	18.10 ± 1.72
*Tiliacora triandra*	Ethanol	> 100	65.30 ± 2.04
	Water	79.48 ± 2.12	21.04 ± 2.09
Chinese ink (Inkstick)	Ethanol	N/A	N/A
	Water	> 100	> 100
*Monetaria moneta* (Money cowry)	Ethanol	N/A	N/A
	Water	> 100	> 100
Mahanintangtong formula	Ethanol	14.86 ± 0.240	15.31 ± 1.01
	Water	34.43 ± 0.47	22.58 ± 1.33
BHT		5.66 ± 0.26	14.87 ± 0.94
Trolox		4.71 ± 0.04	5.02 ± 0.20

Means of three measurements ± SEM (*n* = 3); BHT and Trolox were used as a positive control for antioxidant activity

## Discussion

In this, the first study to examine the properties of Mahanintangtong, we have shown that it possesses good anti-inflammatory activity and good antimicrobial activity, especially against *S. pyogenes*, an important cause of illnesses like pharyngitis [23].

In Thai traditional medicine, symptoms and diseases are thought to be due to an imbalance of the hot and cold elements in body and the carbonized herbs of the Mahanintangtong formula are used to reduce Pitta (fire element) for illnesses like aphthous stomatitis, measles, chicken pox in children up to 12 years old [12, 24]. Research can substantiate the scientific bases for using traditional medicines and bring to light important compounds that could, with future development, be used in patients. This was the rationale of our study in which we used 6 common ingredients and 5 carbonized herbs i.e. *E. rheedii*, *S. pinnata*, *S. rarak*, *C. caesius*, and *T. grandis*.

The extraction methods and solvents used are important in the processing of the bioactive constituents because plants contain a complex blend of phytochemicals [25]. Decoction and 95% EtOH maceration are standard methods and we used a modification of the Thai traditional maceration method. Extract yields depend on pharmacognosy of the different parts of the plants used. The water extracts from the leaves were double those of the ethanol extracts whereas most of the EtOH extracts from wood were higher than the water extracts. For the seeds, the water extract of carbonized *E. rheedii* and *S. pinnata* seeds was higher and lower than the alcohol extracts, respectively. However, yields of water and ethanol extracts of Mahanintangtong formula were similar.

The ethanol extracts of all herbs and Mahanintangtong had greater antibacterial activity for the gram-positive (+) and gram-negative (−) bacteria compared to the water extracts, especially against *S. pyogenes*; the carbonized ethanol extracts had the highest activity. Carbonization is done because it is believed to result in better tolerability but might reduce their efficacy. Never the less, our carbonized ethanol extract retained high anti streptococcal activity. Moreover, the carbonized ethanol extracts of *E. rheedii*, *S. pinnata*, *S. rarak* and *T. grandis* had greater activity against the Gram-positive bacteria compared to *P. aeruginosa*. Similar results have been reported by Sameh et al., 2019 [26] who reported that the essential oil from *S. pinnata* was highly active against MRSA and *S. aureus*. This oil contains sesquiterpenes, β-caryophyllene, oxygenated monoterpenes and α-terpineol are the main constituent and it is noteworthy that β-caryophyllene has been shown to have potent activity against *S. aureus*, *P. aeruginosa* with MICs of 3 ± 0.4 and 7 ± 1.2 μM [27]; α-terpineol also has anti-*S. pyogenes* activity [28]. By contrast, Bitchagno et al. [29] demonstrated that the ethanol extract from the fruit of *T. grandis* showed good activity against the Gram-negative bacteria, especially *E. coli*.

The reduced anti Gram-negative activity in our study is probably due to their hydrophilic cell wall structure which is essentially made up of LPS that is impermeable to hydrophobic oils and so prevents accumulation in their cell membranes [30]. *C. albicans* was resistant to all extracts and this may be related to the its ability to produce extracellular enzymes that degrade and metabolize substrates on its surface. Therefore, the presence of plant extracts are a source of food to the fungi rather than inhibiting their growth but we also used a low concentration of plant extract which may part explain poor inhibition of *C. albicans* [31].

The ethanol extract of Mahanintangtong formula showed good anti-inflammatory activity by inhibiting the release of NO, TNF-α and IL-6 and supports its use by in Thai traditional medicine. Moreover, previous research demonstrates the anti-inflammatory effect of the roots of *P. cablin*, a key component of Mahanintangtong, by inhibiting TNF-α, IL-1β and IL-6 in rats with experimentally-induced palmar oedema [32]. The aerial parts of *P. cablin* also inhibited TNF-α in a human promonocytic cell line (U937 cell) and a human colonic adenocarcinoma cell line (HT-29 cells) [33]. A major component of patchouli oil from *P. cablin* is β-patchoulene that inhibits pro-inflammatory cytokines such as TNF-α, IL-1β, IL6, PGE2 and NO; it also suppresses malondialdehyde, a marker of oxidant stress, and myeloperoxidase, a key enzyme in the respiratory burst of neutrophils [34]. Patchoulene epoxide which isolated from patchouli oil also inhibits TNF-α, IL-1β, IL-6, PGE2 and NO and downregulates mRNA expression of COX-2 and inducible nitric oxide synthetase [35]. Another important component, the ethanol extract from the bark of *T. grandis*, also inhibits TNF-α in RAW 264.7 cells and immortalized mouse splenic dendritic cells [36]. Phytochemicals present in *T. grandis* include naphthoquinone, anthraquinone, monoterpene, diterpene, triterpene, apocarotenoid, phenolic compounds and flavonoids, steroids/saponins, phenylpropanoids and fatty esters [37]. Lapachol, a naphthoquinone, has anti-inflammatory properties in a rat model [38] as do the anthraquinones rubiadin [39] and obtusifolin [40]. Other research has shown that the methanol extract of *D. loureiroi* has antipyretic properties using the brewer’s yeast induced fever method [41]. Taken together, these data show clearly that different key constituents of Mahanintangtong formula possess anti-inflammatory properties and support the use of Mahanintangtong formula in Thai traditional medicine.

We used the DPPH and ABTS^+^ assays to assess antioxidant activity of our extracts. Although they share the same mechanism of antioxidant action i.e. electron transfer, DPPH radicals are more suitable for lipophilic antioxidants while ABTS+ radicals can react with both hydrophilic and lipophilic antioxidants. We demonstrated the antioxidant activities of *E. rheedii*, *S. pinnata*, *T. grandis* and *D. loureiroi*, consistent with previous research. Rheediinosides A and B from *E. rheedii* [42] and the ethanol extract of *D. loureiroi* have been shown to possess potent antioxidant activity [43] whilst *S. pinnata* decreased significantly the levels of antioxidant enzymes [44] and showed similar antioxidant activity as in our study [45].

In 1999, the list of herbal medicine in the NLEM was announced. Mahanintangtong is one of herbal formulas on the list. However, there are no scientific reports on the formula. The present study is the first one to emphasize the antimicrobial, anti-inflammatory and antioxidant activities of Mahanintangtong formula extract. We focused on in-vitro pharmacological activity against sore throat. Our findings suggest that the ethanol of Mahanintangtong formula extract could be used to decrease inflammation, and it also showed anti-bactericidal action, especially *S. pyogenes*. This research supports Thai traditional medicine wisdom, and it can promote the use of Mahanintangtong in hospitals and primary health care units. However, both the quality and quantity of herbal raw materials are one of the main challenges for a Thai herbal product industry. This is in contrast with Western medicine that uses chemical drugs. Thus, the variation between different production batches is limited. In our next research, we would like to study the quality control of the chemical constituents of Mahanintangtong formula. Also, the evidence-based in vivo and clinical practice should be studied so that it is possible that the Mahanintangtong will be developed in biofilm formation.

## Conclusion

This study has brought to light the antimicrobial, anti-inflammatory and antioxidant activities of the Mahanintangtong formula and its constituent herbs. The Mahanintangtong ethanol extract had significant anti *S. pyogenes* activity and significant anti-inflammatory activity that was due to *D. loureiroi*. These results support the use of Mahanintangtong in Thai traditional medicine for minor febrile illnesses and additional development work for possible future clinical use outside of traditional medicine.

## Data Availability

The datasets used and/or analyzed during the current study are available from the corresponding author on reasonable request.

## Abbreviations

ABTS: 2,2′-Azino-bis (3-ethylbenzo-thiazoline-6-sulfonic acid) diammonium salt
BHT: Butylated hydroxytoluene
DMEM: Dulbecco’s modified eagle medium
DMSO: Dimethylsulfoxide
DPPH: 2, 2 –Diphenyl-1-picrylhydrazyl
EtOH: Ethanol
FBS: Fetal bovine serum
HCl: Hydrochloric acid
IL-6: Interleukin 6
LPS: Lipopolysaccharide from *E. coli* O55:B5
MBC: Minimum bactericidal concentration
MHA: Mueller-Hinton agar
MHB: Mueller-Hinton broth
MIC: Minimum Inhibitory Concentration
MRSA: Methicillin resistant *Staphylococcus aureus*
MTT: Thiazolyl blue tetrazolium bromide
NO: Nitric oxide
OD: Optical density
PBS: Phosphate buffer saline
RAW 264.7: Mouse macrophage leukemia-like
SRB: Sulphorhodamine B
TNF-α: Tumor necrosis factor α
